# Complement Receptors 1 and 2 in Murine Antibody Responses to IgM-Complexed and Uncomplexed Sheep Erythrocytes

**DOI:** 10.1371/journal.pone.0041968

**Published:** 2012-07-25

**Authors:** Christian Rutemark, Anna Bergman, Andrew Getahun, Jenny Hallgren, Frida Henningsson, Birgitta Heyman

**Affiliations:** 1 Department of Medical Biochemistry and Microbiology, Uppsala University, Uppsala, Sweden; 2 Department of Immunology, University of Colorado Denver School of Medicine and National Jewish Health, Denver, Colorado, United States of America; University of Ottawa, Canada

## Abstract

Early complement components are important for normal antibody responses. In this process, complement receptors 1 and 2 (CR1/2), expressed on B cells and follicular dendritic cells (FDCs) in mice, play a central role. Complement-activating IgM administered with the antigen it is specific for, enhances the antibody response to this antigen. Here, bone marrow chimeras between *Cr2^−/−^* and wildtype mice were used to analyze whether FDCs or B cells must express CR1/2 for antibody responses to sheep erythrocytes (SRBC), either administered alone or together with specific IgM. For robust IgG anti-SRBC responses, CR1/2 must be expressed on FDCs. Occasionally, weak antibody responses were seen when only B cells expressed CR1/2, probably reflecting extrafollicular antibody production enabled by co-crosslinking of CR2/CD19/CD81 and the BCR. When SRBC alone was administered to mice with CR1/2^+^ FDCs, B cells from wildtype and *Cr2^−/−^* mice produced equal amounts of antibodies. Most likely antigen is then deposited on FDCs in a way that optimizes engagement of the B cell receptor, making CR2-facilitated signaling to the B cell superfluous. SRBC bound to IgM will have more C3 fragments, the ligands for CR1/2, on their surface than SRBC administered alone. Specific IgM, forming a complex with SRBC, enhances antibody responses in two ways when FDCs express CR1/2. One is dependent on CR1/2^+^ B cells and probably acts via increased transport of IgM-SRBC-complement complexes bound to CR1/2 on marginal zone B cells. The other is independent on CR1/2^+^ B cells and the likely mechanism is that IgM-SRBC-complement complexes bind better to FDCs than SRBC administered alone. These observations suggest that the immune system uses three different CR1/2-mediated effector functions to generate optimal antibody responses: capture by FDCs (playing a dominant role), transport by marginal zone B cells and enhanced B cell signaling.

## Introduction

Complement is central to a well functioning immune defense. Its most well known role is to induce lysis of target cells which occurs when the end product of complement activation, the membrane attack complex, forms pores in the target cell membrane. Complement is also important in the initiation of inflammation and in elimination of circulating immune complexes. Less well known is that complement is crucial for antibody responses to both thymus dependent and thymus independent antigens (reviewed in [1], [2], [3]) as first demonstrated when mice depleted of C3 by treatment with cobra venom factor proved to have a severely impaired antibody response [4]. Subsequently, humans and animals genetically deficient in C1, C2, C3, and C4 were found to have impaired antibody responses, especially to suboptimal concentrations of antigen [5], [6], [7], [8], [9], [10]. Lack of C1q [5], [11], but not of factor B in the alternative pathway [12], [13] or of MBL in the lectin pathway [14], [15] leads to impaired antibody responses, suggesting that the classical pathway is of major importance. One of the most efficient activators of the classical pathway is IgM antibodies. Specific IgM, passively administered to mice prior to certain antigens such as erythrocytes, keyhole limpet hemocyanine, and malaria parasites, enhances the antibody responses to these antigens [16], [17], [18], [19], [20]. The enhancing effect of IgM depends on its ability to activate complement [20], [21] but enhancement is unperturbed in mice lacking the lytic pathway of complement activation owing to lack of factor C5 [21], excluding that increased lysis of antigen explains the immunostimulatory effect of IgM. Instead, ability to enhance correlates with deposition of C3 fragments on the surface of the IgM-complexed antigen [11].

Mice lacking CR1/2 owing to gene targeting [22], [23], [24] or to antibody blockade [25], [26] have a similar phenotype as that observed in mice lacking the soluble complement factors C1, C3, or C4, i e impaired primary and secondary antibody responses. The role of CR1/2 is more pronounced with low antigen doses [25], [26]. C1 and C4 are required to form classical C3 convertase which cleaves C3 into the split products C3b, iC3b, C3dg, and C3d which are the ligands for CR1/2. Therefore it seems likely that the impaired antibody responses seen in the absence of C1, C3, C4, and CR1/2 are all caused by lack of CR1/2-mediated effects, either indirectly owing to failure to cleave C3 and generating the receptor ligands or directly by the absence of CR1/2. Murine CR1/2 are derived from the same gene (*Cr2*) by alternative splicing and *Cr2^−/−^* mice therefore lack both receptors [27], [28].

Although it is well established that CR1/2 are of crucial importance for antibody responses, the mechanism by which they operate is not well understood. B cells and/or FDCs must be involved since they are the only cell types expressing the receptors in mice [29], and hypotheses involving either B cells or FDCs have been put forward. An obvious role for FDCs would be to capture antigen-complement complexes and present them to B cells in primary follicles and germinal centers, thus facilitating class switch recombination and affinity maturation. Involvement of CR1/2^+^ B cells could have several explanations. First, co-crosslinking of the BCR and the CR2/CD19/CD81 complex in vitro can lower the threshold for B cell activation [30], [31], [32] and this may take place also in vivo. A second possibility is that B cells transport antigen-complement complexes from the marginal zone into the B cell follicles in a complement dependent manner. Marginal zone B cells express high levels of CR1/2, shuttle between the marginal zone and the splenic follicles [33], and have been shown to transport IgM-complexed antigen to FDCs [34]. In lymph nodes, CR1/2^+^ B cells play a role in transporting IgG-complexed antigen to FDCs [35], [36]. Finally, the possibility that B cells take up antigen-complement complexes via CR1/2 and present antigenic peptides to T helper cells, thus inducing a more efficient antibody response, has been discussed. This mechanism operates in vitro [37], [38] but induction of T helper cells in vivo is equally efficient in wildtype and *Cr2^−/−^* mice [39], [40], [41] suggesting that lack of antigen presentation by T helper cells does not explain the impaired antibody responses in *Cr2^−/−^* mice. Murine, as well as human, CR1 can serve as a cofactor for factor I-mediated cleavage of C3b [42], [43]. This could possibly lead to increased deposition of C3d fragments on antigens, thus further potentiating any of the proposed effector mechanisms discussed above.

To understand how CR1/2 exert their role in immune responses, it is important to elucidate whether expression on B cells or on FDCs is required. Previous work, studying antibody responses to antigen administered alone, has given different results. In some studies, expression on FDCs plays a pre-dominant role [41], [44], [45] whereas in other studies B cell expression is most important [22], [46]. To our knowledge, the relative role of these receptors on B cells and FDCs in responses to IgM-antigen complexes has never been investigated. The aim of the present study was to determine the role of CR1/2 on B cells and FDCs in responses to SRBC administered alone as well as together with specific IgM. To this end, bone marrow chimeras between wildtype and *Cr2^−/−^* mice were generated and immunized with SRBC or with IgM anti-SRBC and SRBC. For a robust IgG anti-SRBC response, expression of CR1/2 on FDCs is required. When FDCs express CR1/2, presence of CR1/2^+^ B cells further increases the response to IgM-SRBC complexes but has no effect on the response to uncomplexed SRBC. When only B cells express CR1/2, weak, often rapidly declining, responses can occur provided sufficiently high doses of SRBC or IgM-SRBC complexes are administered. The data presented suggest that the immune system utilizes CR1/2 in several ways in responses to one and the same antigen.

## Materials and Methods

### Mice

BALB/c mice were from Bommice (Ry, Denmark). Mice lacking CR1/2 (*Cr2^−/−^*) [23] were backcrossed for 10 generations to BALB/c and absence of CR1/2 expression was confirmed by PCR and flow cytometry as described earlier [47]. Ig allotype congenic mice, C.BKa-*Igh^b^*/IcrSMnJ (CB17), were obtained from The Jackson Laboratory (Bar Harbor, Maine, USA). All mice were bred and maintained in the animal facilities at the National Veterinary Institute (Uppsala, Sweden). Animals were age and sex matched within each experiment and all animal experiments were approved by Uppsala Animal Research Ethics Committee (Permit numbers: C117/7 and C146/10).

### Antigens

SRBC were purchased from the National Veterinary Institute (Håtunaholm, Sweden) and stored at 4°C in sterile Alsever's solution. Erythrocytes were washed in PBS three times before use.

### Immunizations and blood sampling

All injections were made in one of the lateral tail veins with the indicated doses of antigen and antibodies in 0.2 ml PBS. When IgM was used, 0.2 ml of a preparation with a hemagglutination titer of 1∶32 was given in PBS one hour prior to immunization with SRBC. Blood sampling was made from the tails or retro-orbital plexa at the indicated time points. Sera were stored at −18**°**C prior to analysis.

### Antibodies

For flow cytometry we used rat IgG2bκ anti-CD16/CD32 (FcγIII/II, clone 2.4G2) (Fc-block), phycoerythrin (PE) labeled rat IgG2aκ anti-CD45R (B220, clone RA3-6B2), biotinylated mouse IgG1κ anti-IgM^a^ mAb (clone DS-1), biotinylated anti-IgM^b^ mAb (clone AF6-78), and fluorescein isothiocyanate (FITC) labeled streptavidin (BD Pharmingen, San Diego, CA). For ELISA we used alkaline phosphatase conjugated sheep anti-mouse IgG or goat anti-mouse IgM, (Jackson ImmunoResearch Laboratories, West Grove, PA), and biotinylated mouse anti-mouse IgG1^a^ (clone 10.9), IgG2a^a^ (clone 8.3), IgG1^b^ (clone B68-2), and IgG2a^b^ (clone 5.7) (BD Pharmingen).

### IgM-purification

Five ml sera from BALB/c mice immunized i.v. five days earlier with 0.2 ml of a 10% SRBC suspension was applied onto a Sepharose-CL 6B (GE-Healthcare, Uppsala, Sweden) column at 0.3 ml/min (Sigma-Aldrich) using PBS containing 0.02% NaN_3_ as buffer. Nine ml fractions were collected and the IgM and IgG protein peaks tested for IgM- and IgG-anti-SRBC in ELISA. Fractions containing IgM (and no IgG) were pooled and concentrated by spin columns (Amicon Ultra Centrifugal Filter Units, NMWL 50 kDa, Millipore, Billerica, MA, USA) at 4000 rpm for 10 min. The supernatants were then filtered through a sterile syringe filter 0.45 µm (VWR, West Chester, PA) and stored at −18°C prior to use.

### Hemagglutination assay

Fifty µl purified IgM was serially diluted in 2-fold steps in PBS in V-bottomed microtiter plates (Greiner Bio-One GmbH, Frickhausen, Germany). Twenty-five µl 1% SRBC suspension in PBS and 25 µl of PBS was added to each well and plates were incubated at 37°C for one hour. The hemagglutination titer was defined as the highest dilution where hemagglutination of SRBC was still detected.

### Irradiation and bone marrow transplantation

Female BALB/c, *Cr2^−/−^* and CB17 mice were whole body irradiated with 7.5 Gy and rested for 24 hours before i.v. transfer of 5 or 10×10^6^ bone marrow cells per mouse in 0.2 ml PBS. Bone marrow cells was prepared from both hind legs of donor mice. Mice were rested for at least six weeks before use in experiments.

### Flow cytometry

For determination of B cell phenotypes in chimeric mice, 200 µl blood from each mouse was collected in 100 µl PBS with heparin (50 U/ml; Leo Pharma AB, Malmö, Sweden) six weeks after bone marrow transplantation. One hundred µl of the cell suspension was transferred to FACS tubes (BD Falcon, BD Biosciences, Bedford, MA, USA) and erythrocytes were removed by lysis in hypotonic buffer [0.15 M NH_4_Cl (Merck), 1.0 mM KHCO_3_ (Sigma-Aldrich), 0.1 mM Na_2_EDTA (Sigma-Aldrich), pH 7.3] for 5 min. Cells were washed twice in PBS containing 2% fetal calf serum, FCS (Sigma-Aldrich) and blocked with anti-CD16/CD32 to prevent unspecific binding. To this, 50 µl biotinylated anti-IgM^a^ or -IgM^b^ antibodies in predetermined optimal concentrations was added and incubated during gentle shaking at 4°C for 30 min. Cells were then washed twice in PBS containing 2% FCS. A mixture of streptavidin-FITC and B220-PE was then added in 50 µl and incubated during gentle shaking at 4°C for 30 min, followed by two washes in PBS containing 2% FCS. The cells were counted on an LSRII or FACScan flow cytometer (BD Biosciences) and analyzed with Flow Jo software (Tree Star, Inc).

### Enzyme-linked immunosorbent assay (ELISA)

The IgG- and IgM-anti-SRBC ELISAs were described earlier [11], [40]. For detection of allotype specific IgG anti-SRBC, a mixture of biotinylated anti-mouse IgG1^a^, IgG2a^a^, or a mixture of biotinylated anti-mouse IgG1^b^, IgG2a^b^, was added and the plates were developed with streptavidin conjugated with alkaline phosphatase (BD Pharmingen). The absorbance was determined at 405 nm after 30 min and data were analyzed with SOFTmax software (Molecular Devices, Sunnyvale. CA, USA). Results are given as OD values in sample dilutions chosen so that high values do not reach a plateau level.

### Statistical analysis

Statistical differences between groups were determined by Student's *t*-test. For allotype specific data, the paired Student's *t*-test was used. Statistical significance levels were set at: ns = p>0.05; * = p<0.05; ** = p<0.01; *** = p<0.001.

## Results

### Expression of CR1/2 on FDCs is required for a robust IgG anti-SRBC response to SRBC

**Figure 1:**
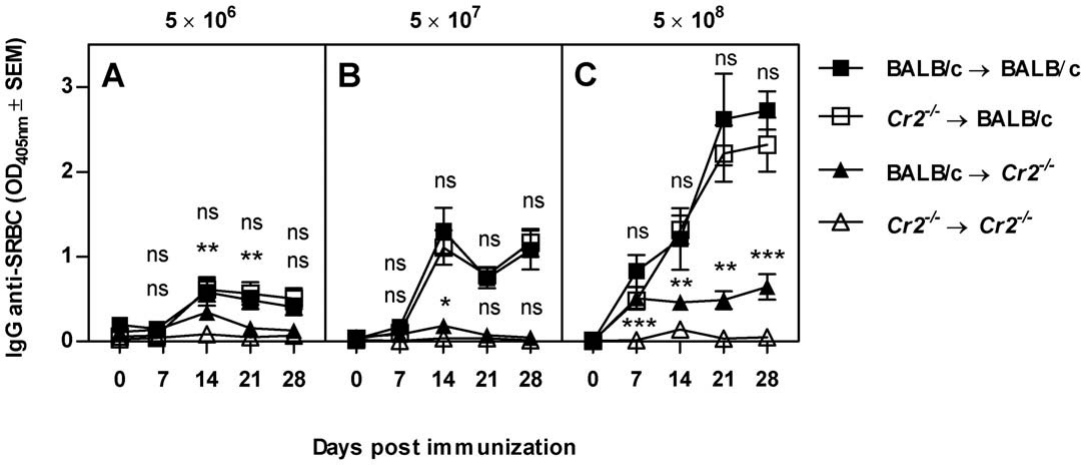
CR1/2 on FDCs are required for a robust IgG anti-SRBC response to SRBC. BALB/c and *Cr2^−/−^* mice were irradiated and reconstituted with either BALB/c or *Cr2^−/−^* bone marrow. Six weeks after reconstitution, mice (n = 6/group) were immunized i.v. with 5×10^6^, 5×10^7^, or 5×10^8^ SRBC. All mice were bled at indicated time points. Sera were diluted 1∶125 (A) or 1∶625 (B and C) and screened for IgG anti-SRBC in ELISA. P-values represent comparisons between the responses in recipients with the same background; ns = p>0.05; * = p<0.05; ** = p<0.01; *** = p<0.001. Representative of two (A) or one (B, C) experiments.

To determine whether expression of CR1/2 on B cells or on FDCs are required for a normal antibody response, bone marrow chimeric mice were generated. Recipients and donors were either wildtype BALB/c or *Cr2^−/−^* mice (on a BALB/c background) resulting in four different phenotypes: CR1/2 on B cells and FDCs (BALB/c→BALB/c), CR1/2 on either B cells (BALB/c→*Cr2^−/−^*) or FDCs (*Cr2^−/−^*→BALB/c) or CR1/2 on neither of the cell types (*Cr2^−/−^*→*Cr2^−/−^*). These animals were immunized with three different doses of SRBC and their IgG responses were analyzed over the next four weeks (Figure 1). As expected, mice lacking CR1/2 both on B cells and FDCs had a poor antibody response. Mice expressing CR1/2 on their FDCs had a robust IgG anti-SRBC response regardless of whether their B cells were derived from BALB/c or *Cr2^−/−^* bone marrow. In mice where only the B cells expressed CR1/2, either no response or a very weak response (to the highest SRBC dose) was seen (Figure 1C).

### B cells from *Cr2^−/−^* and wildtype mice produce similar amounts of IgG anti-SRBC to SRBC

**Figure 2:**
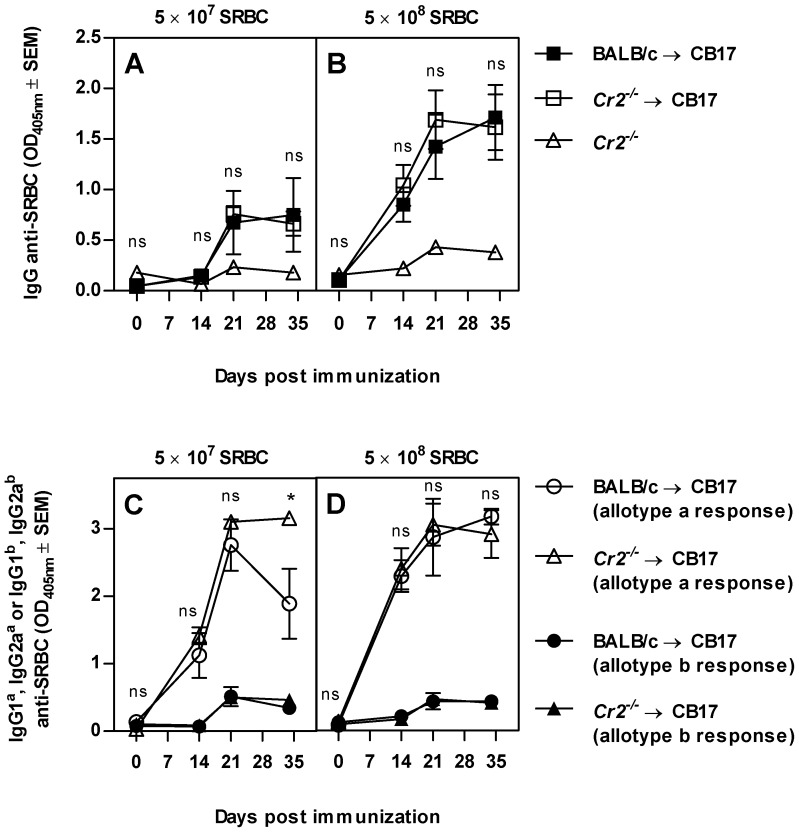
Wildtype and *Cr2^−/−^* B cells produce similar amounts of IgG anti-SRBC. CB17 (Ig^b^ allotype) mice were irradiated and reconstituted with either BALB/c or *Cr2^−/−^* bone marrow (both Ig^a^ allotype) (n = 6/group). Six weeks after reconstitution, chimeras and *Cr2^−/−^* (n = 4, as negative control) mice were immunized with 5×10^7^ (A and C) or 5×10^8^ SRBC (B and D) i.v. All groups were bled at indicated time points. Sera were screened for total IgG anti-SRBC (A and B; diluted 1∶320) and for SRBC-specific IgG1 and IgG2a of the a and b allotype (C and D; diluted 1∶40). P-values represent comparisons between the responses in mice transplanted with BALB/c and *Cr2^−/−^* bone marrow; ns = p>0.05; * = p<0.05; ** = p<0.01; *** = p<0.001. Representative of one experiment.

It appeared from the data described above that B cells from BALB/c and *Cr2^−/−^* mice produced similar amounts of IgG when CR1/2^+^ FDCs were present (compare curves with filled and open squares, Figure 1). To rule out that the IgG detected in (*Cr2^−/−^*→BALB/c) chimeras was in fact produced by wildtype B cells remaining in the recipient mice in spite of the irradiation, Ig allotype chimeras were generated. CB17 is a BALB/c mouse strain congenic for the Ig locus, producing antibodies of the Ig^b^ allotype whereas BALB/c and *Cr2^−/−^* mice produce Ig^a^ antibodies. Bone marrow from either BALB/c or *Cr2^−/−^* was transferred to CB17 recipients and after six weeks the chimeras were immunized with SRBC. The total IgG anti-SRBC response, measured in an ELISA detecting all IgG allotypes, was similar whether the B cells were of *Cr2^−/−^* or BALB/c origin (Figure 2A, B). *Cr2^−/−^* mice, used as a negative control, produced very low levels of IgG anti-SRBC (Figure 2A, B, open triangles). In an ELISA detecting only SRBC-specific IgG1 and IgG2a of the Ig^a^ (donor) allotype, B cells from wildtype and *Cr2^−/−^* mice produced similar amounts of IgG (Figure 2C, D, open symbols) except at day 35 after immunization with 5×10^7^ SRBC, when *Cr2^−/−^* -derived B cells in fact produced more IgG (Figure 2C). Very little IgG anti-SRBC of the recipient allotype (Ig^b^) was detected, showing that B cells from wildtype recipients did not contribute significantly to the antibody response (Figure 2 C, D, filled symbols).

**Figure 3:**
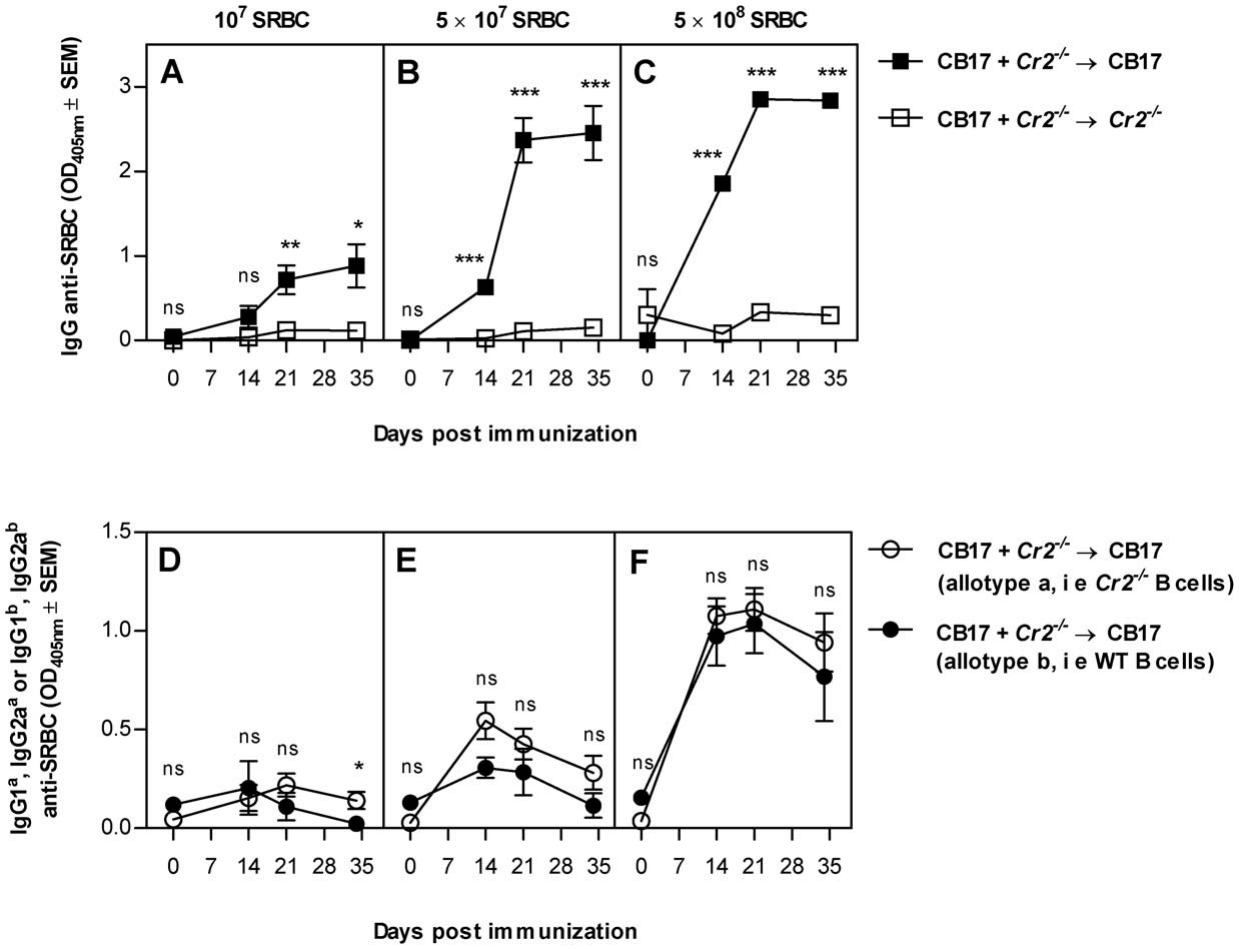
Wildtype and *Cr2^−/−^* B cells produce similar amounts of IgG anti-SRBC in the same mouse. CB17 (Ig^b^ allotype) and *Cr2^−/−^* (Ig^a^ allotype) mice were irradiated and reconstituted with a mixture of CB17 and *Cr2^−/−^* bone marrow, resulting in mice having both CR1/2 positive and negative B cells but either expressing CR1/2 on FDCs (n = 6–8) or not (n = 6–8). Six weeks after transplantation, mice were immunized with 1×10^7^ (A and D), 5×10^7^ (B and E) or 5×10^8^ (C and F) SRBC i.v. All groups were bled at indicated time points. Sera were diluted 1∶40 and screened for total IgG anti-SRBC (A–C) or for IgG1 and IgG2a of Ig^a^ and Ig^b^ allotypes (D–F). P-values were calculated with Student's *t*-test and represent comparisons between the responses in mice expressing CR1/2 on FDCs or not (Student's *t*-test; A–C), and between the different allotypes within the same mouse (paired Student's *t*-test, D–F), where ns = p>0.05; * = p<0.05; **,  = p<0.01; ***,  = p<0.001. Representative of three experiments.

In all the experiments described above, B cells from *Cr2^−/−^* and wildtype mice were operating in separate animals. To be able to compare the antibody production by these two types of B cells within the same mouse, mixed chimeras were generated. Recipients were CB17 or *Cr2^−/−^* mice and each mouse received equal amounts of CB17 and *Cr2^−/−^* bone marrow. Therefore, all mice had a mixed B cell compartment and FDCs which either expressed or did not express CR1/2. This system should minimize the influence of environmental factors and B cells with and without CR1/2 will also compete for antigen under equal terms. Six weeks after bone marrow reconstitution, the B cell compartment in the chimeras was analyzed in flow cytometry using antibodies that distinguished between B cells of Ig^a^ and Ig^b^ allotypes. Both donor strains contributed similarly to the B cell pool with an average of 45% B cells with IgM^a^ (CR1/2^−^) and 55% with IgM^b^ (CR1/2^+^) allotype. As expected, upon immunization *Cr2^−/−^* recipients produced little or no IgG anti-SRBC (Figure 3A–C). In contrast, CB17 recipients produced high titers of total IgG anti-SRBC (Figure 3A–C). Notably, B cells from *Cr2^−/−^* and CB17 mice produced similar titers of SRBC-specific IgG1 and IgG2a, measured as Ig^a^ and Ig^b^ allotypes respectively (Figure 3D–F). In conclusion, mice lacking CR1/2 on their FDCs are unable to produce significant amounts of IgG anti-SRBC. In the presence of CR1/2^+^ FDCs, B cells from *Cr2^−/−^* and wildtype mice produce equal amounts of specific IgG as tested by three different experimental approaches (Figures 1, 2, 3).

### Expression of CR1/2 on both FDCs and B cells is required for an optimal antibody response to IgM-SRBC complexes

**Figure 4:**
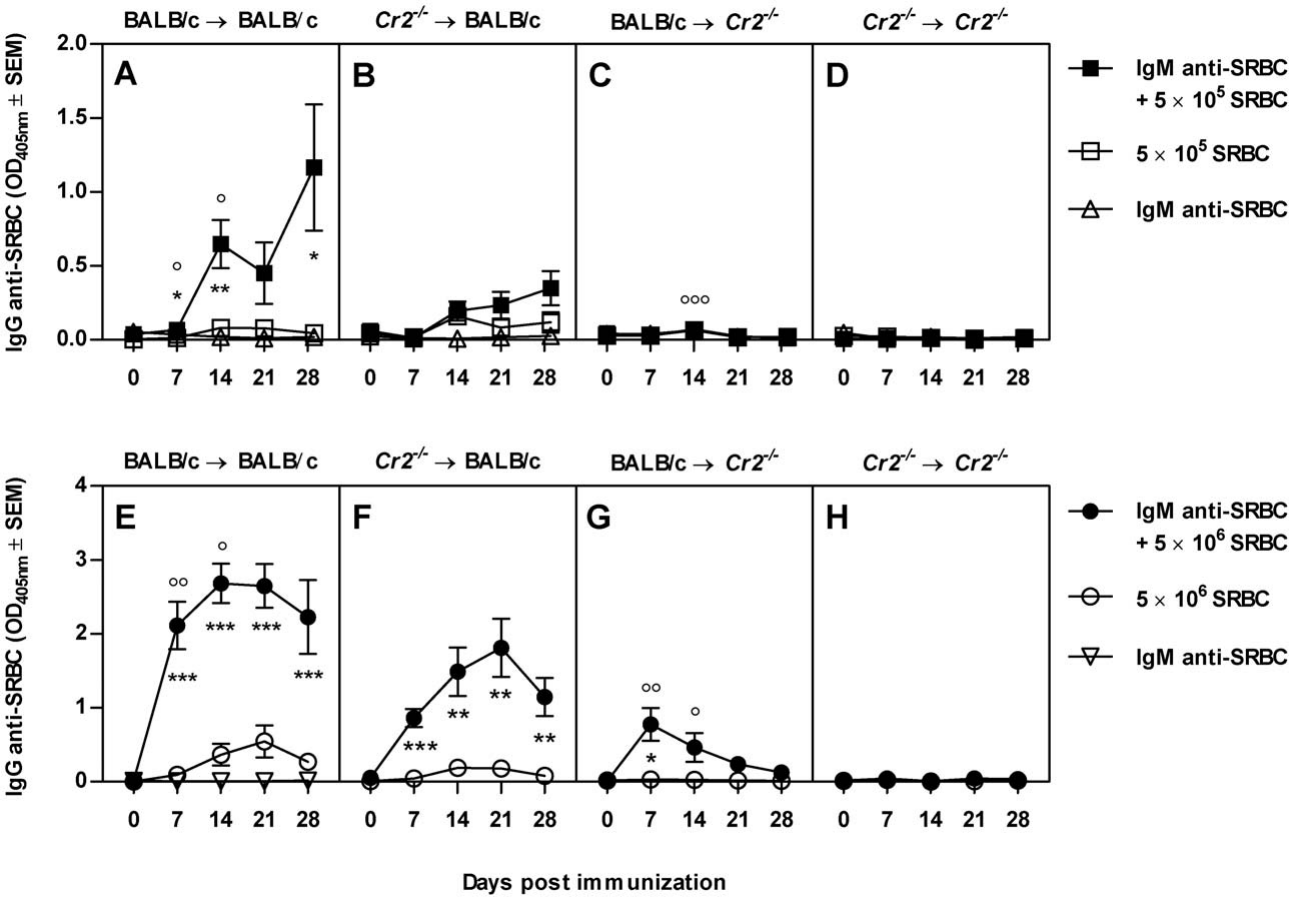
CR1/2 on B cells and FDCs is required for optimal antibody responses to IgM-SRBC complexes. BALB/c and *Cr2^−/−^* mice were irradiated and reconstituted with either BALB/c or *Cr2^−/−^* bone marrow. Six weeks after transplantation, mice were immunized with 5×10^5^ (A–D) or 5×10^6^ (E–H) SRBC alone (open squares) or together with IgM anti-SRBC with a hemagglutination titer of 1∶32 (filled squares) or with IgM anti-SRBC alone (open triangles) (n = 6/group). All mice were bled at indicated time points. Sera were diluted 1∶25 (A–D) or 1∶625 (E–H) and screened for IgG anti-SRBC. Two statistical comparisons were made, both using Student's *t*-test. First, comparisons between the responses in mice immunized with SRBC alone versus IgM and SRBC (to determine whether IgM enhanced antibody responses significantly; filled versus open symbols), where ns = p>0.05; * = p<0.05; ** = p<0.01; *** = p<0.001. Second, comparisons between the responses between various chimeras immunized with IgM-SRBC (to determine whether CR1/2^+^ B cells contributed significantly to the antibody response to IgM-SRBC in mice with CR1/2^+^ FDCs (A vs B; E vs F) and CR1/2^−^ FDCs (C vs D; G vs H)), where ns = p>0.05; ° = p<0.05; °° = p<0.01; °°° = p<0.001. For graphic clarity, non-significant differences are not indicated. Representative of one (A–D) and two (E–H) experiments.

The observation that CR1/2 expression on B cells did not play a substantial role for antibody responses to SRBC was surprising. CR1/2 on B cells are important for B cell signaling in vitro [30], [31], for the transport of IgG-antigen complexes from the subcapsular sinus into lymph node follicles [35], [36], and for the transport of IgM-antigen complexes into the spleen follicles [34], [48]. As mentioned in the introduction, antigen-specific IgM administered with its antigen can feedback enhance the antibody response to this antigen [16], [18], [20], [21]. IgM-mediated enhancement is dependent on the ability of IgM to activate complement [20], [21] and does not operate in C3-depleted or *Cr2^−/−^* mice [21], [47]. We hypothesized that responses to IgM-SRBC complexes may be more dependent on CR1/2 expression on B cells than are responses to SRBC alone. To test this, bone marrow chimeras were immunized with SRBC alone, IgM anti-SRBC alone, or IgM anti-SRBC together with SRBC. In these experiments, suboptimal doses of SRBC were used since IgM does not enhance against high doses of antigen [16], [49]. As expected, IgM was able to enhance the response to both 5×10^5^ and 5×10^6^ SRBC in (BALB/c→BALB/c) chimeras (Figures 4A, E), whereas no enhancement took place in (*Cr2^−/−^*→*Cr2^−/−^*) chimeras (Figures 4D, H). Interestingly, the antibody response to IgM-SRBC complexes was higher in (BALB/c→BALB/c) than in (*Cr2^−/−^*→BALB/c) chimeras (cf Figures 4A with 4B and 4E with 4F). This shows that expression of CR1/2 on B cells, in addition to FDCs, is required for an optimal IgG response to IgM-SRBC and thus differs from what was seen after immunization with SRBC alone where CR1/2^+^ B cells were not required for an optimal response (Figures 1 and 3). The role of B cells was most pronounced in responses to the lowest dose of SRBC, where a response to IgM-SRBC complexes was barely detectable without the presence of CR1/2^+^ B cells (Figures 4B). Another interesting finding was that IgM efficiently enhanced the antibody response in the absence of CR1/2^+^ B cells, provided CR1/2^+^ FDCs were present (Figure 4F). A weak IgG anti-SRBC response was observed in (BALB/c→*Cr2^−/−^*) chimeras immunized with IgM-SRBC (Figure 4G), resembling what was seen after immunization with SRBC alone (Figure 1C). In summary, IgM enhances antibody responses in several ways. Two are dependent on CR1/2^+^ B cells: one is seen when FDCs express (Figure 4 A,E) and the other when FDCs do not express (Figure 4G) CR1/2. The third way is independent on CR1/2^+^ B cells and is seen in mice where only FDCs express CR1/2 (Figure 4F).

## Discussion

**Figure 5:**
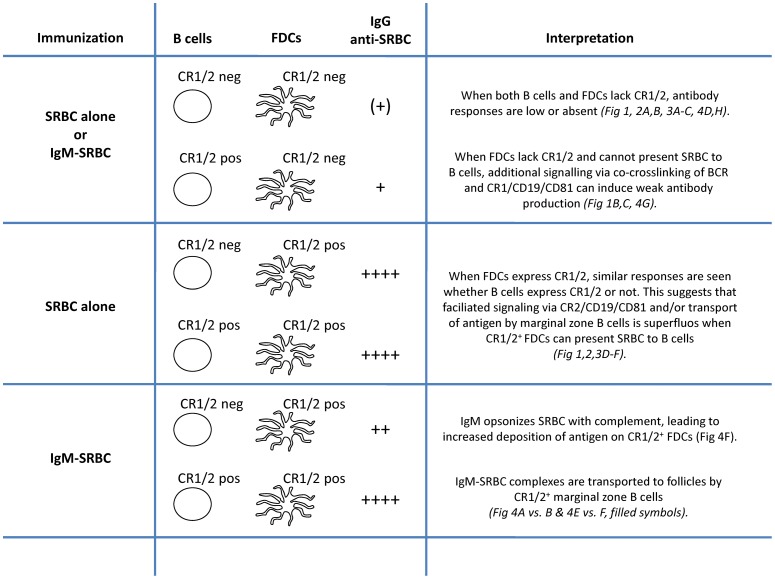
Antibody responses in chimeric mice after immunization with SRBC alone or IgM anti-SRBC+SRBC.

Here, the complicated question of how CR1/2 contribute to the antibody response to IgM-complexed as well as to uncomplexed SRBC has been addressed. Focusing on the participation of the two CR1/2-expressing cells, B cells and FDCs, it was found that the immune system utilizes these receptors in several ways to optimize immune responses. The “default” pathway, by far playing the most significant role, is that complement-opsonized SRBC are captured by CR1/2^+^ FDCs which efficiently present the antigen to B cells. In addition, our data suggest that CR1/2^+^ B cells in some situations contribute to an optimal antibody response. A summary of these findings is presented in Figure 5.

### Responses to SRBC or IgM-SRBC when FDCs lack CR1/2 (Figure 5, top)

The crucial role for CR1/2 in antibody responses shown previously [22], [23], [24], [25], [26], [44] was confirmed because IgG responses both to SRBC alone and to IgM-SRBC were severely impaired when both B cells and FDCs lacked CR1/2. As a rule, CR1/2^+^ FDCs were required for robust IgG responses. However, weak responses both to SRBC and IgM-SRBC were occasionally detected when FDCs lacked CR1/2. This required that CR1/2^+^ B cells were present (Figure 1C, Figure 4G) and most likely represent extrafollicular antibody production [50]. It can be envisaged that when the FDCs lack CR1/2, they do not display antigen to B cells in an optimal way, and therefore appropriate crosslinking of BCRs cannot take place. In this situation it is feasible that only B cells where the threshold for activation can be lowered by co-crosslinking of the BCR and the CR2/CD19/CD81 co-receptor complex will be triggered to antibody production [30], [31], [32]. Thus, when FDCs lack CR1/2, the requirements for CR1/2 expression for antibody responses were similar whether mice were immunized with SRBC or IgM-SRBC: lack of CR1/2 on both cells led to severely impaired responses whereas expression of CR1/2 on B cells only resulted in weak (extrafollicular) responses.

### Responses to SRBC when FDCs express CR1/2 (Figure 5, middle)

In responses to SRBC alone, CR1/2^+^ FDCs were always required for robust IgG anti-SRBC responses and in their presence B cells were equally efficient producers of IgG anti-SRBC whether they expressed CR1/2 or not. These conclusions are based on observations in three different types of chimeric mice, where contribution of recipient B cells and B cell extrinsic factors was carefully excluded (Figures 1, 2, 3). A likely explanation for why CR1/2 on B cells are superfluos in the presence of CR1/2^+^ FDCs, is that antigen captured by FDCs can be concentrated to a high density and presented in an efficient way to the B cells. This results in crosslinking of a sufficient number of BCR without the facilitated signaling by co-crosslinking to CR2/CD19/CD81. In a previous study, CR1/2^+^ B cells were shown to contribute to the IgG anti-SRBC responses in mice immunized with SRBC alone, also in the presence of CR1/2^+^ FDCs [44]. The mice used in that study were on a different genetic background than our mice. A possible explanation for the difference is that FDCs in our mice are more efficient in capturing opsonized SRBC, and therefore do not need contribution of B cells. It seems likely that the crucial role for CR1/2^+^ FDCs in antibody responses is explained by their ability to capture complement-coated SRBC, thereby initiating the germinal center reaction known to be important for class switch recombination and IgG production. In support of this, IgG-responses are more dependent on CR1/2 than are IgM responses [11], [23] and formation of normal germinal centers requires the presence of CR1/2 [22], [41]. In order to reach the FDCs, the antigen must be transported to the follicles from the marginal zone where intravenously administered antigens initially end up. An interesting question is how this transportation takes place. To our knowledge, there are three defined pathways by which antigen enters splenic follicles: (i) via marginal zone B cells which shuttle between the marginal zone and the follicles [33] and which have been shown to transport IgM-antigen complexes [34], (ii) via follicular B cells which capture IgE-complexed antigen on their low affinity Fc-epsilon-receptor, CD23, in peripheral blood and transport the complexes to the splenic follicles [51], and (iii) via small channels, conduits, which transport antigens smaller than 60 kD [52]. Since SRBC are too large to gain access to conduits, this route of transportation is very unlikely to occur. Our finding that antibody responses to SRBC administered alone are equally strong when B cells lack CR1/2 as when they express the receptors, provided CR1/2^+^ FDCs are present, exclude a transportation route dependent on CR1/2 on B cells. However, it does not exclude that marginal zone B cells, or follicular B cells, transport SRBC bound to other receptors than CR1/2. For example, natural IgM could bind to SRBC and the complex attach to the FcmuR expressed both on marginal zone and follicular B cells [53] or to CD22 [54]. This scenario would also explain why the impaired antibody response reported in mice lacking secretory IgM [55], [56] does not depend on the ability of IgM to activate complement [11].

### Responses to IgM-SRBC when FDCs express CR1/2 (Figure 5, bottom)

The role of CR1/2 on B cells versus FDCs in responses to IgM-SRBC has not been studied before, and we here made the interesting observation that IgM acts in two ways when CR1/2^+^ FDCs are present. One way is dependent on CR1/2^+^ B cells and is evidenced by the higher antibody responses in chimeras expressing CR1/2 on their FDCs when also B cells expressed CR1/2 (Figure 4 A,B,E,F). This finding leads to the question how the CR1/2^+^ B cells act to enhance responses to IgM-SRBC. An effect on B cell signaling through co-crosslinking of BCR and CR2/CD19/CD81 seems unlikely, since this did not play a role in responses to SRBC alone, once the FDCs expressed CR1/2 and could present antigen efficiently to B cells (Figures 1, 2, 3). Instead, we favour the idea that IgM-SRBC-complement complexes bind to CR1/2 on marginal zone B cells which transport them into the follicles as previously described for KLH-IgM complexes [34], [48]. This interpretation is compatible with the observations that IgM-mediated enhancement of antibody responses is dependent on the ability of IgM to activate complement [20], [21] and that it is paralleled by an increased antigen concentration in the spleen [17]. The other way by which IgM enhanced responses in mice is independent of CR1/2^+^ B cells. This is evidenced by the marked enhancement in chimeras where FDCs, but not B cells, expressed CR1/2 (Figure 4F). The likely explanation is that IgM which binds to SRBC induces massive deposition of C3 fragments on the surface of the antigen. Since C3 fragments are the ligands of CR1/2, IgM-SRBC will be more efficiently captured by CR1/2^+^ FDCs than SRBC administered alone which have less C3 fragments on their surface [11]. The findings in Figure 4F illustrate, in analogy with what was seen with SRBC administered alone, that IgM-SRBC reach the follicle via an unkown route which does not require binding to CR1/2 on B cells.

The overall quality of an antibody response is dependent on many factors such as magnitude, rapidity, affinity, class and subclass distribution. We have here focused on analyzing the total SRBC-specific IgG production and its dependence on CR1/2 on B cells versus FDCs. Overall, the data suggest that SRBC administered alone become opsonized with complement factors which enables FDCs to capture the antigen via CR1/2 after it has been transported from the marginal zone to the splenic follicle. Without CR1/2^+^ FDCs there will be no robust IgG anti-SRBC response. Two major questions remain. First, how does the transport take place if not by binding to CR1/2 on B cells? The data clearly show that responses to SRBC are normal despite the absence of CR1/2^+^ B cells, thus excluding this possibility. Second, how do SRBC become opsonized when they are administered alone? *C1qA^−/−^* mice have impaired antibody responses to SRBC [5], [11], suggesting involvement of classical pathway activation. In spite of this, antibody responses are normal in the absence of the known endogenous classical pathway activators SIGN-R1, SAP, and CRP and in mice whose IgM cannot activate complement [11], leaving open the question of what activates C1q. For antibody responses to SRBC administered together with specific IgM, three different ways to enhance antibody responses could be distinguished based on their requirements for CR1/2 expression on FDCs versus B cells. One operates when only B cells express CR1/2, probably reflecting facilitated B cell signaling, and the other two operate when FDCs express CR1/2. Of these, one requires CR1/2^+^ B cells and probably involves transport of IgM-SRBC from the marginal zone to the follicle. The other was independent of CR1/2^+^ B cells and a possible mechanism is that higher concentrations of C3 fragments are deposited on IgM-SRBC than on SRBC alone, thus increasing the ability of CR1/2^+^ FDCs to capture the antigen once it reaches the follicle. Thus, the data presented suggest that the immune system utilizes CR1/2 in three different ways in responses to SRBC. It is feasible that the relative contribution of these pathways varies depending on which antigen is used and that this, at least in part, may explain the discrepant results regarding the cellular requirements for CR1/2 that have been reported [22], [41], [44], [45], [46].
